# miR-212-5p inhibits nasopharyngeal carcinoma progression by targeting METTL3

**DOI:** 10.1515/med-2022-0515

**Published:** 2022-07-12

**Authors:** Hongyu Zhou, Nana Zhang

**Affiliations:** Department of Otorhinolaryngology Head and Neck Surgery, Wuhan Fourth Hospital, Wuhan 430033, Hubei, China

**Keywords:** NPC, miR-212-5p, METTL3, proliferation, apoptosis

## Abstract

This study was conducted to investigate the effect of microRNA-212-5p (miR-212-5p) on the proliferation and apoptosis of nasopharyngeal carcinoma (NPC) cells. Microarray datasets (EXP00394 and EXP00660) were downloaded from the dbDEMC database, and the differentially expressed microRNAs between high-grade and low-grade NPC were analyzed. miR-212-5p and methyltransferase like 3 (METTL3) expression levels in NPC tissues and cells were determined by the quantitative real-time polymerase chain reaction and Western blot. Besides, the relationship between miR-212-5p expression and clinicopathological characteristics of patients was analyzed by the Chi-square test. Cell counting kit-8 assay, 5-ethynyl-2-deoxyuridine (EdU) assay, and flow cytometry were adopted to detect the effect of miR-212-5p on the cell proliferation and apoptosis. Kyoto Encyclopedia of Genes and Genomes and Gene Ontology analysis were performed to explore the potential biological functions and the signal pathways related to the target genes of miR-212-5p. Bioinformatics prediction and dual luciferase reporter gene assay were used to verify the relationship between miR-212-5p and METTL3 3′ untranslated region. Besides, western blot was adopted to detect the expression of METTL3. Gene set enrichment analysis was performed to analyze the downstream pathways in which METTL3 was enriched. It was found that miR-212-5p was downregulated in NPC tissues, and the low miR-212-5p expression was associated with lymph node metastasis and poor differentiation. miR-212-5p overexpression inhibited the growth and promoted apoptosis of NPC cells; miR-212-5p inhibition functioned oppositely. Mechanistically, miR-212-5p inhibited the proliferation and promoted apoptosis of NPC cells via suppressing METTL3 expression. miR-212-5p/METTL3 was associated with processes of RNA transport and cell cycle. In conclusion, miR-212-5p inhibits the progression of NPC by targeting METTL3.

## 1 Introduction

Nasopharyngeal carcinoma (NPC) is a tumor derived from the nasopharyngeal epithelium and has a higher morbidity and mortality among head and neck malignancies [1,2]. Despite NPC’s sensitiveness to radiotherapy, the prognosis of NPC patients is poor, and the 5-year survival rate of NPC patients is reported to be less than 50% [3,4]. It is, therefore, pivotal to elaborate on the molecular mechanism of the progression of NPC to explore new therapeutic targets for NPC.

MicroRNAs (miRNAs, miRs) are a category of noncoding RNAs that induce the degradation or inhibit the translation of mRNAs by interacting with the 3′ untranslated region (3′UTR) of these mRNAs [5]. miRNAs can act as tumor promotors or inhibitors to regulate the proliferation, differentiation, and apoptosis of cells [6,7]. For example, miR-4321-5p expression is reduced in NPC, and miR-4321-5p overexpression represses the migration and invasion of NPC cells via targeting *N*-myristoyltransferase 1 [8]. miR-124-3p expression is inhibited in NPC. miR-124-3p overexpression curbs the activation of phosphatidylinositol 3-kinase/AKT/mammalian rapamycin target signal pathway by targeting PCDH8 and inhibits the viability and colony formation of NPC cells [9]. As reported, as one of the important members of the miR family, miR-212-5p is dysregulated in a variety of human tumors, such as breast cancer, colorectal cancer (CRC), and clear cell renal cell carcinoma cells, and is associated with the growth and metastasis of tumor cells [10,11,12]. Here, bioinformatics predicted that miR-212-5p expression is inhibited in high-grade tumor tissues of NPC patients; however, how miR-212-5p functions in NPC is unclear.

Previous studies have shown that methyltransferase like 3 (METTL3) promotes the malignant biological behaviors of NPC cells [13,14]. In this study, bioinformatics showed that METTL3 may be a target of miR-212-5p. This study focused on how miR-212-5p functions on the malignant phenotype of NPC cells and its downstream mechanisms.

## 2 Materials and methods

### 2.1 Bioinformatics analysis

Microarray datasets available from dbDEMC database were selected according to following selection criteria. Inclusion criteria were expressed as follows: human case and control study, miRNA expression profile, and complete raw data. EXP00394 and EXP00660 were obtained and analyzed in this study. Differentially expressed miRNA analysis was performed with the “limma” package in R language (version 3.5.2). The adjusted *P* < 0.05 and |log_2_fold change| > 1 were chosen as the cutoff value. With the data in LinkedOmics database, gene set enrichment analysis (GSEA) for METTL3 was conducted to predict its biological functions in NPC. Pathways with NES > 1, nominal *P* value < 0.05, and false discovery rate *q* value < 0.25 were screened out.

### 2.2 Collection of tissue samples

Forty-five patients who admitted to Wuhan Fourth Hospital from June 2016 to April 2019 and diagnosed with NPC by the Department of the Pathology were included, and tumor tissues and adjacent noncancerous tissues were collected during biopsy. All samples were frozen in liquid nitrogen immediately and stored at −80°C for further use. All subjects did not receive chemotherapy, radiotherapy, and other anticancer treatments before the biopsy. This research was approved by the Ethics Committee of Wuhan Fourth Hospital, and all participants signed an informed consent form.

### 2.3 Cell culture and transfection

NPC cell lines (CNE1, 5-8F, C666-1, 6-10B) and the normal nasopharyngeal epithelial cell line NP69 were available from the Cancer Institute of Southern Medical University (Guangzhou, China). All cells were cultured in Roswell Park Memorial Institute-1640 medium with 10% fetal bovine serum (Gibco, Carlsbad, CA), 100 U/mL penicillin (Gibco), and 0.1 mg/mL streptomycin (Gibco) at 37°C in 5% CO_2_ and 95% relative humidity, with the medium changed every 3 days. When cells reached 80–90% confluence, cells were trypsinized with 0.25% trypsin (Roche, Basel, Swizerland). Then, the cells were inoculated into six-well plates at the density of 1 × 10^5^ cells/ml and incubated for 24 h at 37°C in 5% CO_2_. miR-212-5p mimic (miR-212-5p: 3′-UCAUUCGUCAGAUCUCGGUUCCA-5′), mimics control (miR-NC: 5′-UUCUCCGAACGUGUCACGUTT-3′), miR-212-5p inhibitor (miR-212-5p-in: 3′-UGGAACCGAGAUCUGACGAAUGA-5′), inhibitors control (miR-in: 5′-UUGUACUACACAAAAGUACUG-3′), empty vector (NC), METTL3 overexpression plasmid (METTL3), small interfering RNA (siRNA) targeting METTL3 (si-METTL3), and siRNA normal control (si-NC) from GenePharma (Shanghai, China) were subsequently transfected into 5-8F and 6-10B cells with Lipofectamine^TM^ 2000 kit (Invitrogen; Carlsbad, CA). A final concentration of 50 nM of miRNA mimic/inhibitor and inhibitors was used.

### 2.4 Quantitative reverse transcription-polymerase chain reaction

Total RNA was extracted by TRIzol reagent (Invitrogen), and reversely transcribed into cDNA by the miScript Reverse Transcription Kit (QIAGEN, GmbH, Hilden, Germany), and then the miScript SYBR Green PCR system (QIAGEN) was used to perform PCR reactions on a Rotorgene 3000 series PCR machine (Corbett Research, Sydney, Australia). The expression levels of miRNA and mRNA were quantified by Rotorgene software. With U6 or GAPDH as internal references, the relative expressions of miR-212-5p and METTL3 mRNA were calculated by the 2^−ΔΔCT^ method. The sequences of primers: miR-212-5p: 5′-ACCTTGGCTCTCTAGACTGCT-3′, 5′-GCAGGGTCCGAGGTATTC-3′; U6: 5′-CTCGCTTCGGCAGCACA-3′, 5′-AACGCTTCACGAATTTGCGT-3′; METTL3: 5′-AAGCTGCACTTCAGACGAAT-3′, 5′-GGAATCACCTCCGACACTC-3′; GAPDH: 5′-CGGAGTCAACGGATTTGGTCGTAT-3, 5′-AGCCTTCTCCATGGTGGTGAAGAC-3′.

### 2.5 Cell counting kit-8 assay

The transfected cells were inoculated into a 96-well cell plate (1,000 cells per well). After 24 h, 48 h and 72 h, 10 μL of CCK-8 reagent (Invitrogen, Shanghai, China) was added into each well and accordingly incubated for 4 h. The values of OD_450nm_ were detected by a microplate reader.

### 2.6 5-Ethynyl-29-deoxyuridine assay

6-10B and 5-8F cells were inoculated into 24-well plates, respectively. The cells were subsequently incubated for 2 h with 5 μmol/L EdU (Beyotime Biotechnology, Shanghai, China) and rinsed with phosphate buffer saline (PBS). The cells were then fixed with paraformaldehyde for 10 min. 200 μL of glycine at 2 mg/mL was added, and then the cells were incubated for 5 min. Next, the cells were incubated with 100 μL of 0.5% TritonX-100, decolorized on a shaker for 10 min, and immersed twice in PBS for 5 min each time. Then the cells were incubated with Apollo and stained with Hoechst. After the cells were washed with PBS, the cells were observed under a fluorescence microscope.

### 2.7 Flow cytometry

For cell cycle analysis, 6-10B and 5-8F cells were fixed in 70% cold ethanol overnight at 4°C. After washing with PBS twice, the cells were incubated in the binding buffer containing 50 μg/mL propidium iodide (PI; BD Bioscience, San Jose, CA), 20 μg/mL RNase A (Sigma) and 0.2% Triton-X100 for 30 min at room temperature in the dark. Then, 2  ×  10^4^ cells were analyzed using a flow cytometer. For cell apoptosis analysis, the cells were incubated with Annexin V staining solution and PI staining solution at room temperature for 30 min at room temperature in the darkness. Then, 2  ×  10^4^ cells were analyzed using a flow cytometer.

### 2.8 Dual luciferase reporter gene assay

METTL3 wild-type sequence containing the miR-212-5p binding site and the mutant sequence without the miR-212-5p binding site were cloned into the pmirGLO vector (Promega, Madison, WI) to construct a wild-type reporter vector (METTL3-WT) and a mutant reporter vector (METTL3-MUT). Besides, METTL3-WT or METTL3-MUT and miR-212-5p mimics or miR-NC were co-transfected into 293 T cells by Lipofectamine™ 2,000 kit. 48 h later, the dual luciferase reporter gene assay system (Promega) was used to detect the luciferase activity. Specifically, the binding intensity of METTL3 to miR-212-5p was expressed by the ratio of the luminescence intensity of renilla luciferase to that of firefly luciferase.

### 2.9 Western blot

Transfected cells were collected and lysed with RIPA lysis buffer (Pierce, Rockford, IL), and the supernatant was collected by high-speed centrifugation. The protein concentrations were measured by the BCA Protein Assay Kit (Rockford, IL). The extracted protein was mixed with the loading buffer, and the sample was heated at 95°C for 10 min. 30 μg protein was separated on polyacrylamide gel by electrophoresis and transferred to polyvinylidene fluoride membranes. The membranes were blocked with 5% bovine serum albumin for 1 h, then incubated with primary antibody anti-METTL3 (ab195352, 1:1,000, Abcam Cambridge, MA) overnight at 4℃. Moreover, the membranes were washed and incubated with secondary antibody goat anti-rabbit IgG H&L (ab97051, 1:2,000, Abcam Cambridge) for 1 h at room temperature. Subsequent to washing membrane three times, the protein bands were visualized by the chemiluminescent reagent ECL (Beyotime, Shanghai, China), with GAPDH as an internal reference. Bio-Rad Gel Dol EZ imager (GEL DOC EZ IMAGER, Bio-Rad, CA) was adopted to image the proteins, and the gray values were analyzed by Image J software.

### 2.10 Statistical analyses

All experiments were performed in triplicate. SPSS 24.0 software was used for statistical analysis, and GraphPad Prism 9 was used to plot the figures of the experimental data. Comparisons between two groups were conducted by Student’s *t*-test, and differences among multiple groups were compared by one-way analysis of variance, with *P* < 0.05 indicating a statistically significant difference.


**Ethics statement:** Our study was approved by the Ethics Review Board of Wuhan Fourth Hospital.

## 3 Results

### 3.1 MiR-212-5p expression was downregulated in NPC

**Figure 1:**
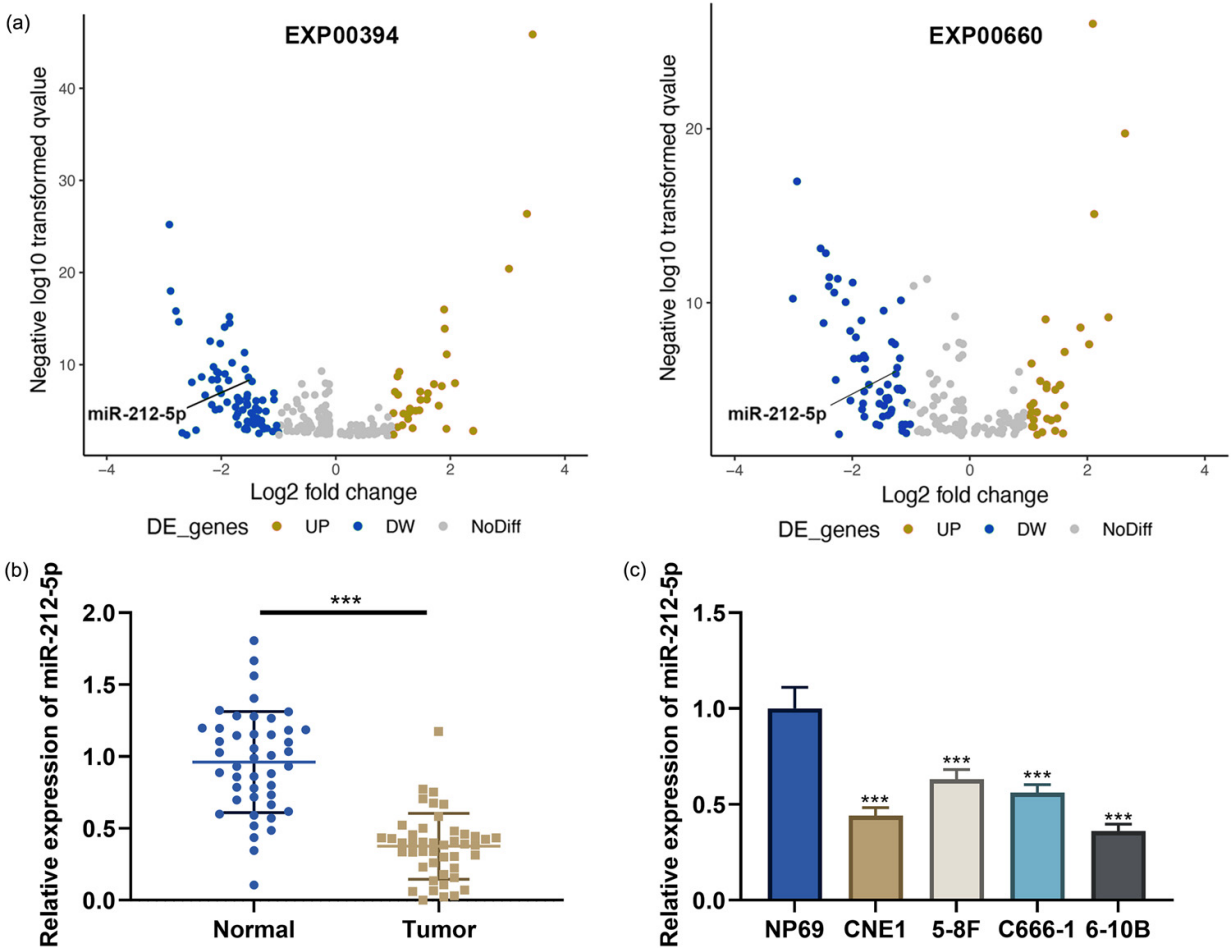
miR-212-5p expression is inhibited in NPC tissues and cells. (a) Volcano plots: microarray datasets EXP00394 and EXP00660 were downloaded from the dbDEMC database to analyze the differential expression of miRNAs between high-grade and low-grade NPC tissues. (b) miR-212-5p expression in NPC tissues and adjacent tissues was detected by qRT-PCR. (c) miR-212-5p expression in human normal nasopharyngeal epithelial cells NP69 cells and NPC cell lines (CNE1, 5-8F, C666-1, 6-10B) was detected by qRT-PCR. ***P < 0.001.

Microarray datasets EXP00394 and EXP00660 were downloaded from the dbDEMC database, and the differentially expressed miRNAs between high-grade tumor tissues and low-grade tumor tissues of NPC patients were analyzed, and it was revealed that miR-212-5p expression was significantly downregulated in high-grade tumor tissues of NPC patients (Figure 1a). Besides, the results of quantitative reverse transcription-polymerase chain reaction (qRT-PCR) highlighted that miR-212-5p expression was significantly inhibited in NPC tissues compared with that in adjacent tissues (Figure 1b). In addition, miR-212-5p was inhibited in four NPC cell lines as against the NP69 cells (Figure 1c). The Chi-square test indicated that low expression of miR-212-5p was closely associated with poorly differentiated tumors and lymph node metastasis in NPC patients (Table 1).

**Table 1:** The correlation between miR-212-5p expression and clinicopathological characteristics of NPC patients

Clinical and pathological indicators	Number	miR-212-5p expression		Chi-square (math.)	*P*-value
		High expression	Low expression		
Age (years)	45	23	22		
≥50	24	12	12	0.025	0.873
<50	21	11	10		
Sex					
Male	23	12	11	0.021	0.884
Female	22	11	11		
Smoking history					
Yes	22	11	11	0.021	0.884
No	23	12	11		
Degree of differentiation					
Moderate and high	24	16	8	4.980	0.026*
Poor	21	7	14		
TNM stage					
I + II	23	15	8	3.746	0.053
III + IV	22	8	14		
Lymph node metastasis					
Yes	20	6	14	6.421	0.011*
No	25	17	8		

Note: *P < 0.05.

### 3.2 Inhibition of miR-212-5p promoted NPC cell viability and restrained the apoptosis

**Figure 2:**
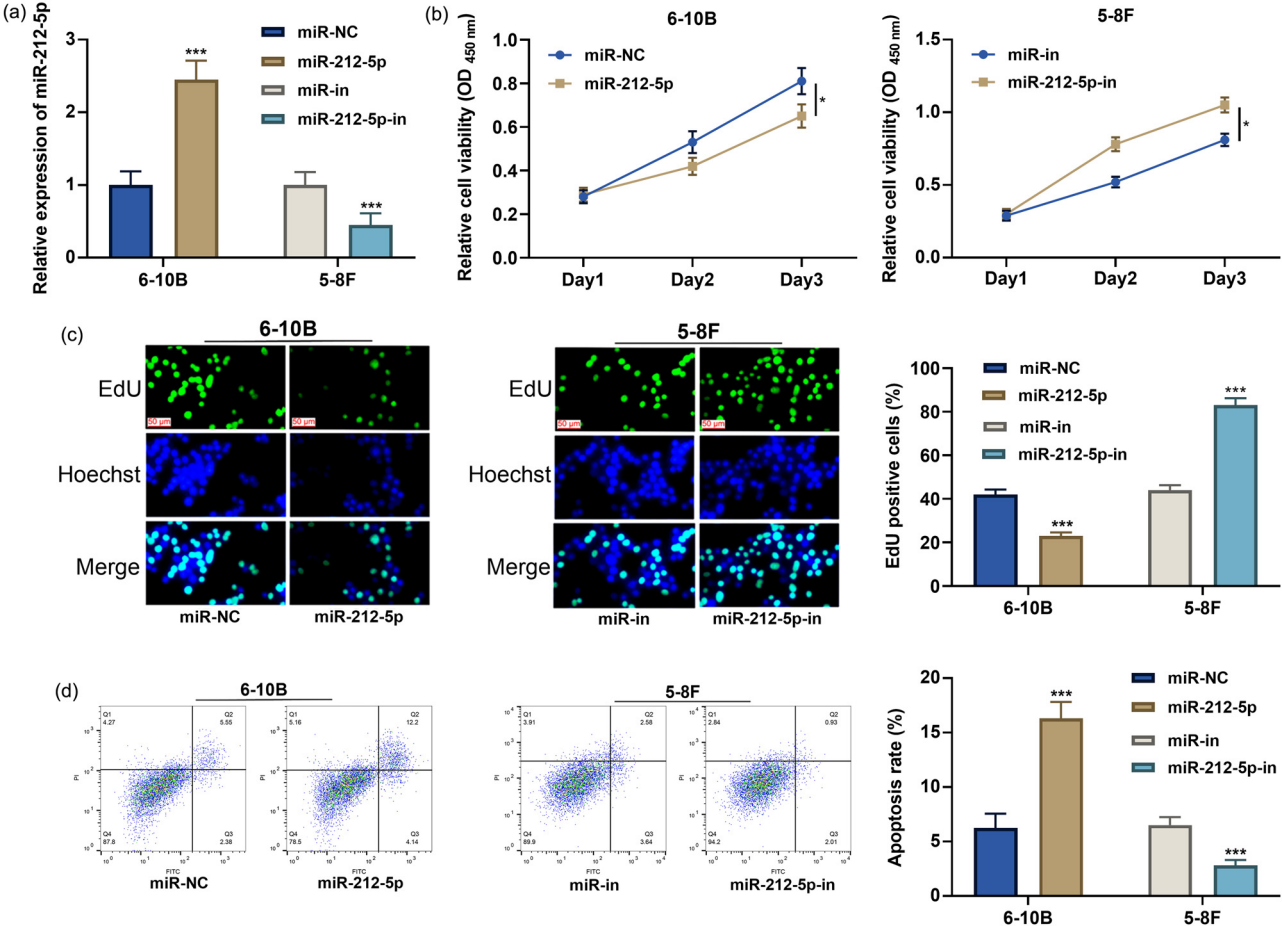
The effect of miR-212-5p on NPC cell proliferation and apoptosis. (a) The expression of miR-212-5p in 6-10B or 5-8F cells transfected with miR-212-5p mimics or inhibitors was detected by qRT-PCR. (b–c) After transfection, the cell proliferation was assessed by the CCK-8 assay and EdU assay. (d) After transfection, the cell apoptosis was assessed by flow cytometry. *P < 0.05, **P < 0.01, and ***P < 0.001.

Subsequently, the function of miR-212-5p in NPC was explored. miR-212-5p mimics were transfected into 6-10B cells, and miR-212-5p inhibitors were transfected into 5-8F cells. qRT-PCR proved the transfection was successful (Figure 2a). CCK-8 assay, EdU assay, and flow cytometry showed that miR-212-5p overexpression restrained the growth and promoted the apoptosis of 6-10B cells compared with the control, while its inhibition worked oppositely on 5-8F cells (Figure 2b–d).

### 3.3 miR-212-5p specifically represses METTL3

**Figure 3:**
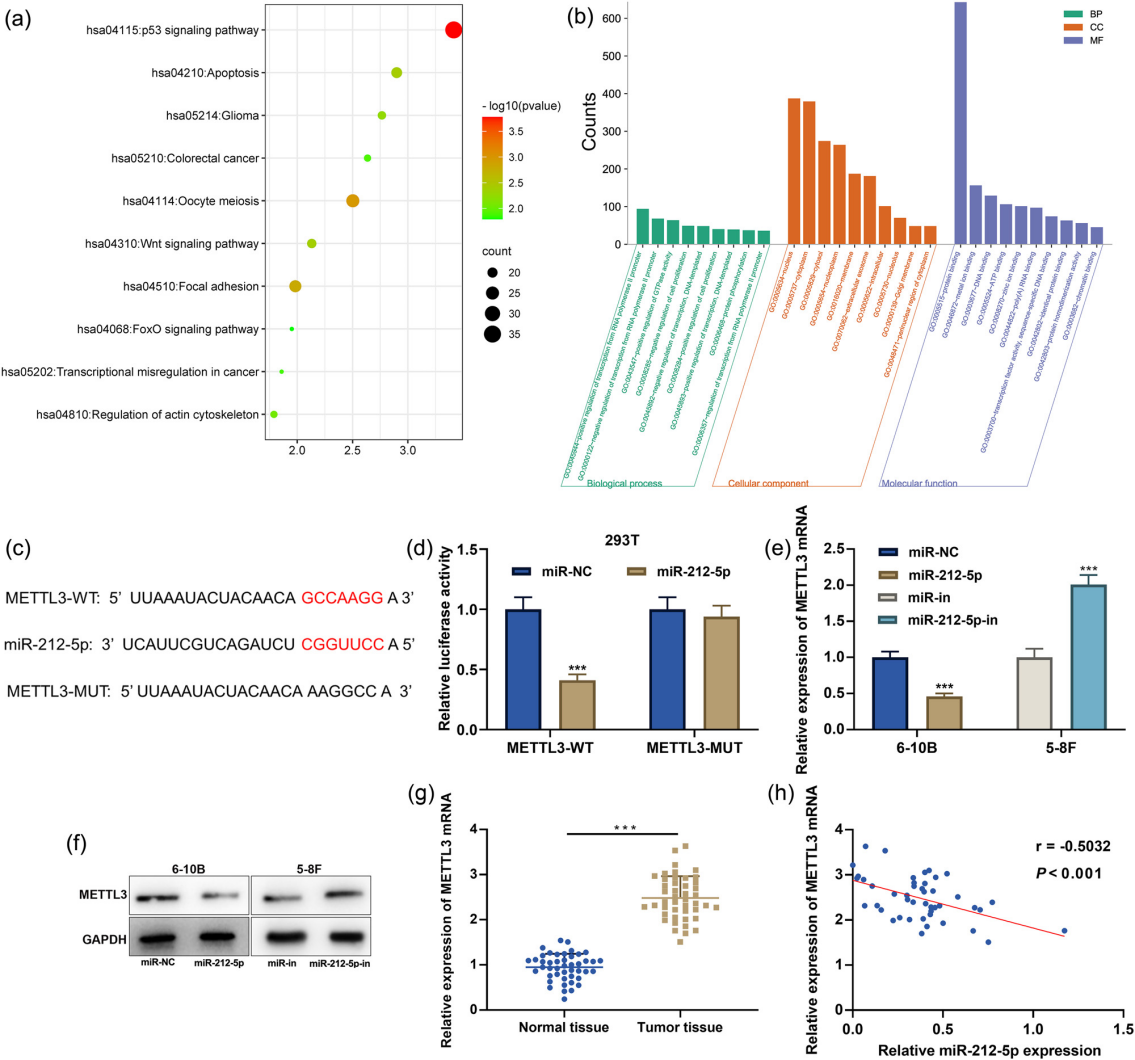
METTL3 is a target of miR-212-5p. (a) The pathway enrichment analysis on the downstream target gene set of miR-212-5p was conducted by the KEGG analysis. (b) The functional enrichment analysis on the downstream target genes of miR-212-5p was conducted by the GO analysis. (c) Binding sequences of METTL3 mRNA 3′UTR wild type or its mutant to miR-212-5p were showed. (d) Dual-luciferase reporter gene assay was used to verify the effect of overexpression of miR-212-5p on the luciferase activity of METTL3-WT and METTL3-MUT. (e and f) The expression of METTL3 mRNA and protein in 6-10B or 5-8F cells transfected with miR-212-5p mimics or inhibitors were detected by qRT-PCR and western blot. (g) The expression of METTL3 mRNA in NPC tissues and adjacent tissues was detected by qRT-PCR. (h) The correlation between METTL3 expression and miR-212-5p expression in NPC tissues was detected by Pearson’s correlation analysis. ***P < 0.001.

miRNA directly binds to the 3′-UTR of a specific mRNA, leading to mRNA degradation and translational repression [15]. The downstream targets of miR-212-5p were predicted by StarBase database and analyzed by Kyoto Encyclopedia of Genes and Genomes (KEGG), and the findings highlighted that the target genes were closely associated with p53 signaling pathway and apoptosis (Figure 3a). Gene Ontology (GO) analysis showed that these targets were mainly enriched in the regulation of cell proliferation and protein phosphorylation in biological processes (BPs); nucleus and cytoplasm in cellular components; and protein binding and metal ion binding in molecular functions (Figure 3b). METTL3 was one of these target genes, and there was a binding site between miR-212-5p and METTL3 mRNA 3′-UTR (Figure 3c). Dual-luciferase reporter gene assay showed that overexpression of miR-212-5p significantly inhibited the luciferase activity of METTL3-WT in 293T cells, but that of METTL3-MUT was not significantly impacted (Figure 3d). qRT-PCR and western blot showed that overexpression of miR-212-5p significantly decreased METTL3 mRNA and protein expression in 6-10B cells, while METTL3 mRNA and protein expressions were greatly increased in 5-8F cells transfected with miR-212-5p inhibitors (Figure 3e and f). In addition, METTL3 mRNA was demonstrably highly expressed in NPC tissues as against that in adjacent tissues (Figure 3g). The correlation analysis showed that miR-212-5p expression in NPC tissues was negatively correlated with METTL3 mRNA expression in NPC tissues (Figure 3h).

### 3.4 miR-212-5p inhibits the proliferation and promotes the apoptosis of NPC cells by targeting METTL3

**Figure 4:**
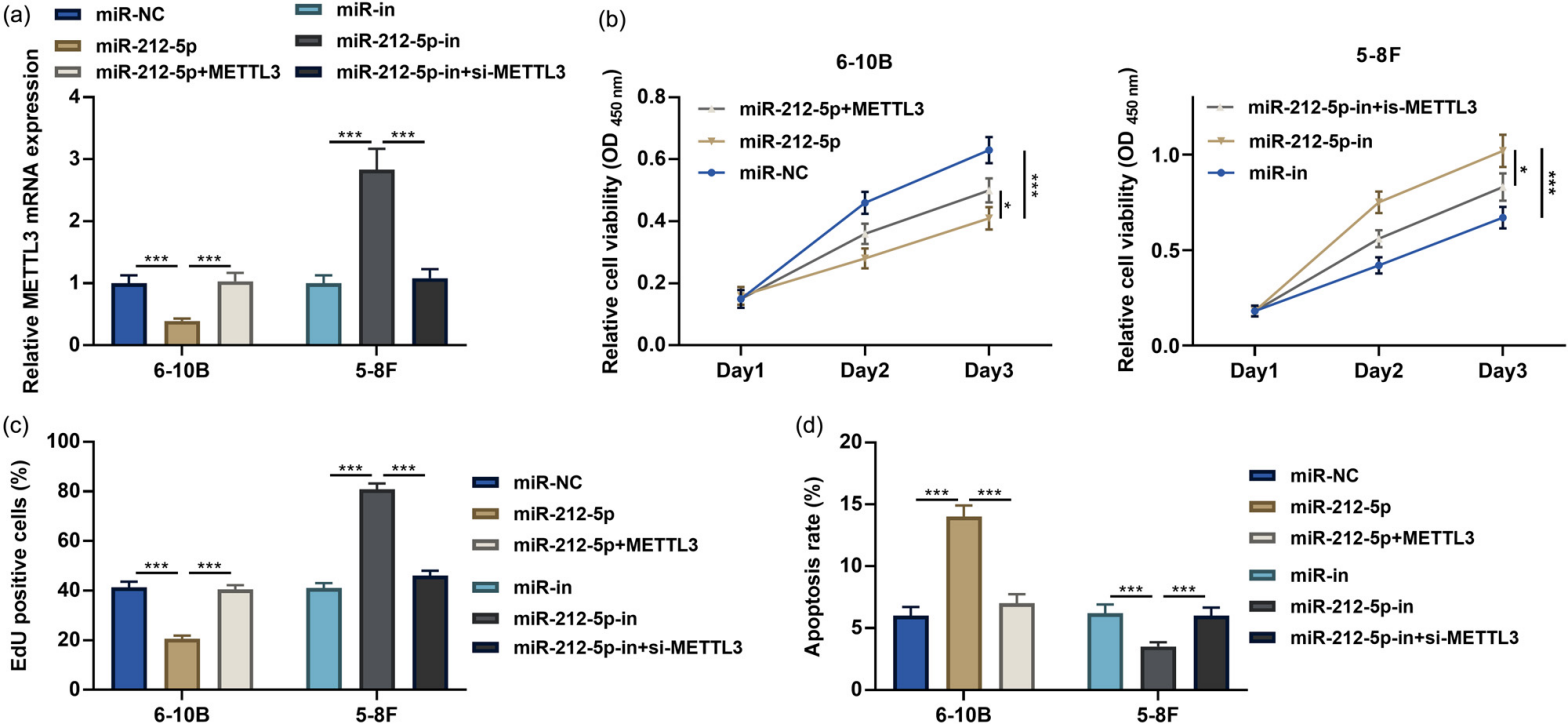
miR-212-5p regulates the proliferation and apoptosis of NPC cells by targeting METTL3. (a) miR-212-5p mimics and METTL3 overexpression plasmids were co-transfected into 6-10B cells, and miR-212-5p-in and si-METTL3 were co-transfected into 5-8F cells. The expression of METTL3 mRNA was detected by qRT-PCR. (b and c) After transfection, the cell proliferation was assessed by the CCK-8 assay and EdU assay. (d) After transfection, the cell apoptosis was assessed by flow cytometry. *P < 0.05, **P < 0.01, and ***P < 0.001.

**Figure A1:**
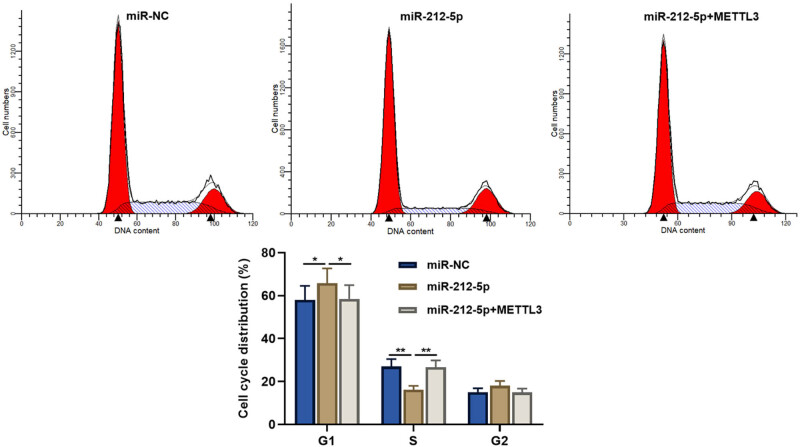
Flow cytometry assay was used to detect the effects of miR-212-5p and METTLE3 on cell cycle progress.

To investigate the function of miR-212-5p/METTL3 axis in NPC, we co-transfected miR-212-5p mimics and METTL3 overexpression plasmids into 6-10B cells, and miR-212-5p inhibitors and METTL3 siRNA into 5-8F cells. qRT-PCR showed that transfection of METTL3 overexpression plasmid could reverse the impact of miR-212-5p overexpression on METTL3 expression; silencing METTL3 counteracted the effects of miR-212-5p inhibition on METTL3 expression (Figure 4a). CCK-8, EdU, and flow cytometry assays proved that miR-212-5p overexpression inhibited the viability and accelerated the apoptosis of 6-10B cells compared with controls, while overexpression of METTL3 reversed these effects (Figure 4b–d); inhibition of miR-212-5p accelerated the growth and inhibited the apoptosis in 5-8F cells, while knockdown of METTL3 impaired these effects (Figure 4b–d). In addition, flow cytometry showed that miR-212-5p overexpression induced the cell cycle arrest, while METTLE3 overexpression accelerated the cell cycle (Figure A1).

### 3.5 miR-212-5p regulates the expression via METTL3

**Figure 5:**
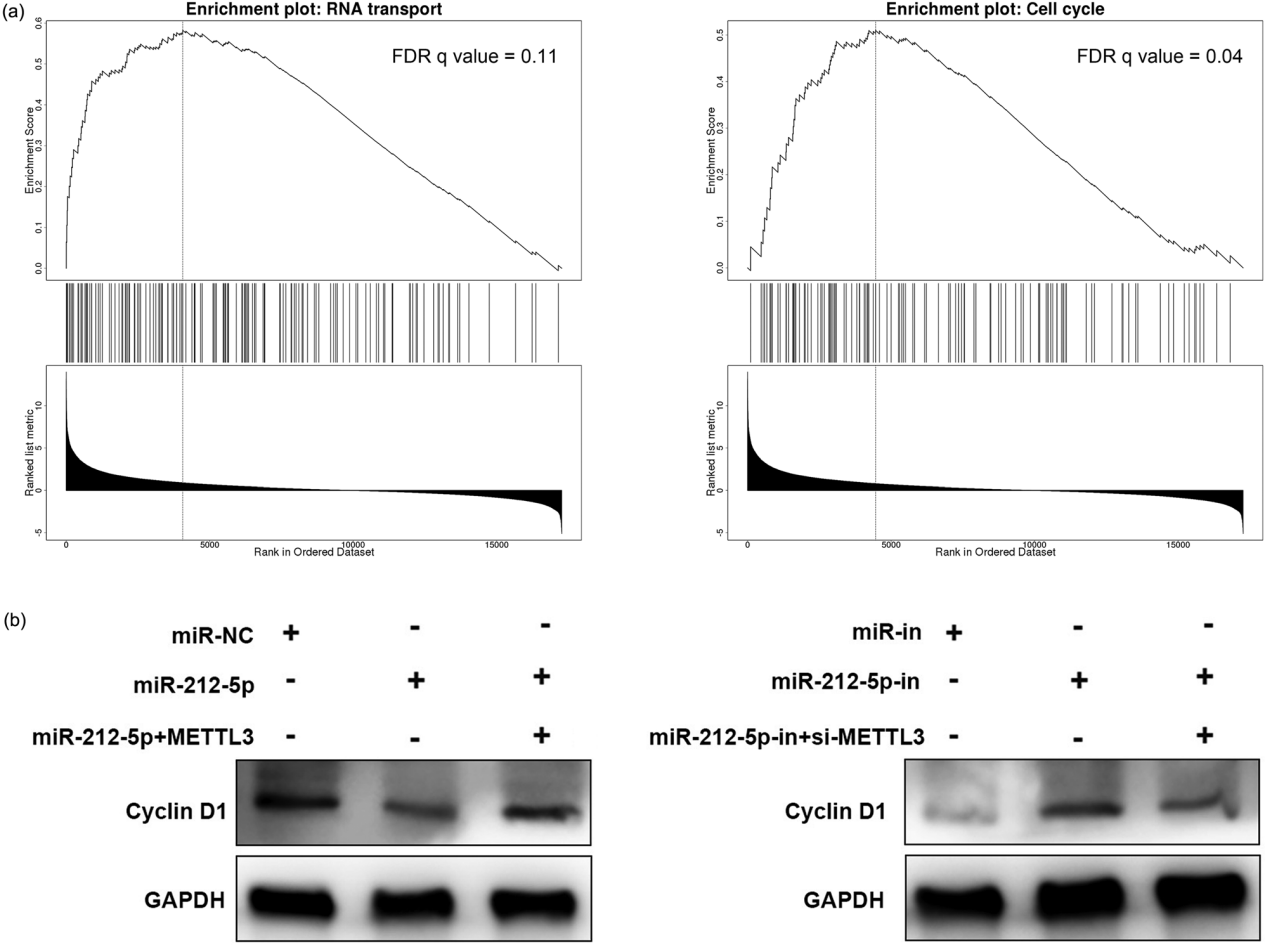
METTL3 activates the DNA replication, cell cycle signaling pathway. (a) Signaling pathway enrichment analysis was conducted through GSEA. (b) miR-212-5p and METTL3 overexpression plasmids were co-transfected into 6-10B cells, and miR-212-5p-in and si-METTL3 were transfected into 5-8F cells. The expression of Cyclin D1 was analyzed by western blot.

We used GSEA for signal pathway enrichment analysis, and the findings suggested that the high expression of METTL3 was associated with the process of RNA transport and cell cycle (Figure 5a). Western blot showed that miR-212-5p overexpression repressed cyclin D1 expression, while overexpression of METTL3 reversed these effects; inhibition of miR-212-5p promoted cyclin D1 expression, while knockdown of METTL3 counteracted these effects. These results further suggested that miR-212-5p inhibited cell cycle progression of NPC cells by regulating METTL3 (Figure 5b).

## 4 Discussion

NPC is mainly distributed in the southern region of China, with obvious geographical and ethnic characteristics; currently, combined radiotherapy and chemotherapy is the main treatment strategy of NPC, and local recurrence and distant metastasis are important reasons for the poor prognosis of NPC patients [16,17]. Early diagnosis and development of treatment therapy are of significance to improve the prognosis of NPC patients [18,19].

miRNAs are a category of noncoding RNAs with a length of 22–24 nt, which can modulate protein expression at posttranscriptional level via combining with the 3′UTR of target mRNAs [20,21,22,23]. miRNAs are widely involved in the regulation of many BP in cancer biology [24]. There is increasing evidence that miRNAs are novel biomarkers and promising therapeutic targets of NPC [25]. Here, we focused on miR-212-5p, which, as reported, is reduced in hepatocellular carcinoma (HCC), and miR-212-5p overexpression restrains the migrative and invasive abilities of HCC cell via targeting the ubiquitin-binding enzyme E2T [26]. miR-212-5p expression is also reduced in CRC tissues and cell lines, and miR-212-5p overexpression represses malignant biological behaviors of CRC cell by targeting SIRT2 [11]. In addition, miR-212-5p expression is also reduced in renal clear cell carcinoma (RCC), and miR-212-5p overexpression blocks the migration, invasion, and proliferation of RCC cells by targeting TBX15 [12]. Here, we demonstrated that miR-212-5p expression was demonstrably decreased in NPC tissues and cells, which was related to clinicopathological indexes in NPC patients. Functionally, miR-212-5p overexpression restrained the growth and promoted the apoptosis of NPC cell; miR-212-5p inhibition exerted the opposite effect. Given our findings, miR-212-5p may function as a tumor suppressor in NPC.

METTL3 is identified as the first m6A methyltransferase [27,28]. Previous studies have shown that METTL3 is functionally complex, and it plays crucial roles in a variety of tissues/cells and is involved in regulating the tumorigenesis and cancer progression [29,30]. Reportedly, high expression of METTL3 is associated with poor prognosis of gastric cancer patients; METTL3 overexpression expedites epithelial–mesenchymal transition process and enhances the migrative and invasive abilities of gastric cancer cells [31]. The upregulated METTL3 facilitates the metastasis of CRC cells via modulating miR-1246/SPRED2/MAPK signaling pathway [32]. In NPC, knockdown of METTL3 inhibits the expression levels of β-catenin/transcription factor 7-like 2 target genes vimentin and N-cadherin and blocks the migration and invasion of NPC cells, implying the oncogenic roles of METTL3 in NPC [13]. Another study reports that METTL3 promotes the migratory and invasive ability of NPC cells by regulating Snail [33]. Also, METTL3 accelerates the progression of NPC through mediating m6A modification of EZH2 [14]. In addition, METTL3 exerts pro-cancer effects in tumors and is also specifically regulated by miRs [34,35,36]. In the present work, we proved that miR-212-5p could target METTL3 to suppress the viability and facilitate the apoptosis of NPC cells.

Considering miR-212-5p is associated with tumor differentiation and lymph node metastasis in the patients, it may be used as a biomarker to predict the prognosis of the patients, and the response to the treatment, and it may also be a potential therapy target for NPC. There are several limitations in this work. First, *in vivo* studies are important to further validate the function of miR-212-5p/METTL3 axis in NPC progression. In addition, whether miR-212-5p/METTL3 axis can regulate other phenotypes of NPC cells (such as chemosensitivity and radiosensitivity) awaits further investigation.
